# Twenty-Two Percent Efficient Pb-Free All-Perovskite Tandem Solar Cells Using SCAPS-1D

**DOI:** 10.3390/nano13010096

**Published:** 2022-12-25

**Authors:** Ali Alsalme, Huda Alsaeedi

**Affiliations:** Department of Chemistry, College of Science, King Saud University, Riyadh 11451, Saudi Arabia

**Keywords:** MAGeI_3_, FAMASnGeI_3_, Pb-free all-perovskite tandem solar cells, tandem solar cells, SCAPS-1D

## Abstract

Herein, we reported the simulation study of lead (Pb)-free all-perovskite tandem solar cells using SCAPS-1D. Tandem solar cells are comprised of two different cells which are known as the top cell and the bottom cell. We simulated tandem solar cells using methyl ammonium germanium iodide (MAGeI_3_) as the top subcell absorber layer due to its wide band gap of 1.9 eV. Further, FA_0.75_MA_0.25_Sn_0.25_Ge_0.5_I_3_ = FAMASnGeI_3_ was used as the bottom subcell absorber layer due to its narrow band gap of 1.4 eV. The tandem solar cells were simulated with MAGeI_3_ as the top cell and FAMASnGeI_3_ as the bottom subcell using SCAPS-1D. Various electro-transport layers (ETLs) i.e., titanium dioxide, tin oxide, zinc oxide, tungsten trioxide, and zinc selenide, were used to examine the impact of ETL on the efficiency of tandem solar cells. The observations revealed that TiO_2_ and ZnSe have more suitable band alignment and better charge-extraction/transfer properties. A reasonably improved efficiency of 23.18% and 22.4% have been achieved for TiO_2_ and ZnSe layer-based tandem solar cells, respectively.

## 1. Introduction

The energy crisis is one of the major challenges for the scientific community which needs significant attention [1,2,3]. Current energy resources are limited, which may be responsible for energy crises in the future [4,5,6]. Hence, it is important to find renewable energy sources to overcome the issue of the energy crisis [7]. Solar energy is one of the most abundant energy sources which can be a suitable candidate for the production of neat and clean energy [8,9,10]. Solar energy can be directly converted and transformed into electrical energy via photovoltaic cells (solar cells) [11]. In the past few decades, various solar cells such as dye-sensitized solar cells, organic solar cells, thin film solar cells, silicon-based solar cells, polymer solar cells, bulk-heterojunction solar cells, quantum dot solar cells, and perovskite solar cells (PSCs) have been developed [3,7,12,13]. At present, silicon-based solar cells are widely used in practical applications but their fabrication process is quite complicated [3]. In addition, silicon-based solar cells are expensive, and it is important to reduce the cost of solar cells [7]. In this connection, a new visible light sensitizer (methyl ammonium lead halide = MAPbX_3_; X = halide anion) was explored in the fabrication of dye-sensitized solar cells [14]. This MAPbX_3_-based dye-sensitized solar cell device exhibited power conversion efficiency (PCE) of less than 4% [14]. However, it was further improved to more than 25% by employing extensive efforts and novel approaches [8]. Unfortunately, a single-junction solar cell has some drawbacks such as sub-bandgap and thermalization losses [15]. Additionally, it has been found that single-junction solar cells cannot exceed the Shockley–Queisser (SQ) single-junction limit proposed by SQ in 1961 [16]. Thus, tandem solar cells have been developed which may be a more suitable and efficient alternative to silicon-based solar cells [15]. In this connection, all-perovskite tandem solar cells have been developed by various research groups which showed decent performance [17,18,19,20,21]. Lead halide-based perovskite materials have been used in the fabrication of tandem solar cells, but the presence of toxic lead (Pb) still remains a concern for their practical applications [19]. In further studies, Pb-free perovskite materials such as methyl ammonium bismuth iodide, methyl ammonium antimony iodide, methyl ammonium tin iodide (MASnI_3_), or methyl ammonium germanium iodide (MAGeI_3_) have been explored in the construction of single-junction PSCs or tandem solar cells [22,23,24,25,26,27,28,29,30]. The reports suggested that Sn- or Ge-based perovskite materials have excellent optoelectronic features and a less toxic nature [30]. Thus, Sn- or Ge-based materials could be the alternative to lead-based perovskite materials [29].

Recently, numerical simulations of single-junction PSCs and tandem solar cells using SCAPS-1D have received enormous attention, and a large number of publications have been reported [31,32,33,34,35,36,37,38]. In this connection, Pandey et al. [37] simulated tandem solar cells by exploring CH_3_NH_3_Pb_0.5_Sn_0.5_I_3_ and Cs_2_AgBi_0.75_Sb_0.25_Br_6_ as the bottom cell and top cell, respectively. The simulated tandem solar cells exhibited a decent short-circuit current density (Jsc) of 15.21 mA/cm^2^ with an excellent open circuit voltage (Voc) of 1.95 V. A high PCE of 21.9% was reported for the simulated tandem solar cell architecture [37]. Further, Madan et al. [38] also simulated tandem solar cells using SCAPS-1D. Madan et al. [38] used FACsPb_0.5_Sn_0.5_I_3_ as the top cell layer and Cs_2_AgBi_0.75_Sb_0.25_Br_6_ as the bottom cell layer. The authors achieved an interesting Jsc of 14.9 mA/cm^2^, Voc of 1.83 V, and PCE of 17.3% using SCAPS-1D. In 2022, MASnI_3_ and MASnIBr_2_ were used as the bottom and top cell materials by Abdelaziz et al. [15]. The authors reported a good PCE of 15.6% which included a Jsc of 13.94 mA/cm^2^ and Voc of 1.89 V. The above results indicate that simulation of tandem solar cells using SCAPS-1D may be useful for the scientific community.

In the present work, our group reports the simulation study on the development of Pb-free all-perovskite tandem solar cells with MAGeI_3_ as the top subcell and FAMASnGeI_3_ as the bottom subcell using SCAPS-1D. The obtained results exhibited the presence of an excellent PCE of 22.4% for the simulated Pb-free all-perovskite tandem solar cells.

## 2. Device Structure and Simulation

Before the investigation of the photovoltaic performance of the all-perovskite tandem solar cell devices, we simulated the device with single-light-absorber layer devices using MAGeI_3_ or FAMASnGeI_3_ with ZnSe as the HTL using Cu_2_O as the HTL. The simulation parameters (band gap, dielectric permittivity, and electron affinity, etc.) and their used values for the simulation studies are presented in Tables S1 and S2. The simulation for all the devices were performed (illumination of AM 1.5 G; 100 mW/cm^2^; temperature = 300 K) using SCAPS-1D software developed by Prof. Marc Burgelman, Belgium [39]. In multi-junction tandem solar cells, two diodes are joined in a series to form the subcells. These joined diodes (subcells) generate the same current which is called the current of the tandem solar cells. Similarly, the sum of the voltage in the subcells is referred to as the voltage of the tandem solar cells. It is well known that SCAPS-1D could not fully support multi-junction tandem solar cells. Thus, the top and bottom subcells were separately simulated. In our simulations, the top subcell was illuminated using standard AM 1.5 G (1 sun conditions) and the bottom subcell was illuminated using the spectra filtered by the top subcell which can be described as below [40]:

(1) $$S\;\left(\lambda \right)=S_{0}\left(\lambda \right).exp\left(\sum_{i=1}^{4}\;-\;ai\left(\lambda \right)di\right)$$

where S(λ) = filtered spectrum; S_0_(λ) = spectrum incident on the top subcell; *a*i(λ) = absorption coefficient; and di is the thickness of the material. Under the above conditions, the Jsc of the two subcells was matched to simulate the tandem solar cells. We have also schematically described the simulation process in Figure S1 in the Supporting Information.

## 3. Results and Discussion

### Photovoltaic Investigations

**Figure 1:**
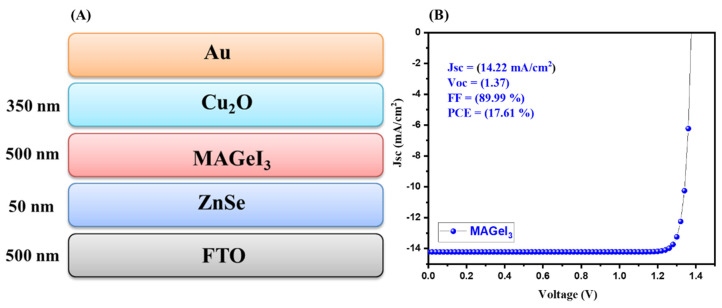
Schematic device structure (**A**) and J–V curve (**B**) of MAGeI_3_-based PSCs.

In the first stage, MAGeI_3_-based solar cells were simulated using SCAPS-1D. The performance of the MAGeI_3_-based solar cells was checked using a short-circuit current density (Jsc)–voltage (V) analysis. A schematic representation of the MAGeI_3_-based solar cell device architecture (FTO(500 nm)/ZnSe(50 nm)/MAGeI_3_(500 nm)/Cu_2_O(350 nm)) is shown in Figure 1A. The collected J–V graph of the simulated MAGeI_3_-based solar cell is presented in Figure 1B. The observations revealed that an excellent open circuit voltage (Voc) of 1.37 V can be achieved for MAGeI_3_-based PSCs with a PCE of 17.61%. In addition, the MAGeI_3_-based simulated device also showed a good Jsc value. The obtained photovoltaic parameters showed the promising performance of MAGeI_3_-based PSCs.

**Figure 2:**
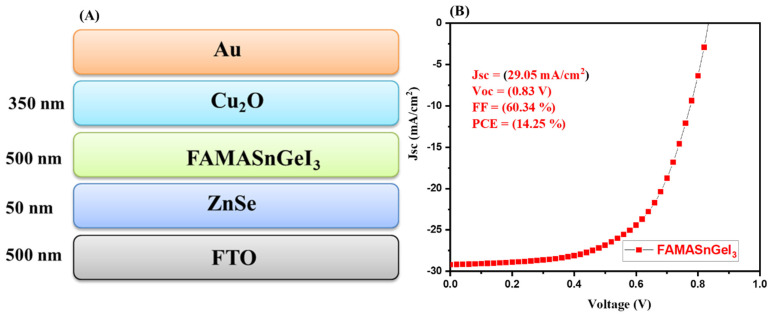
Schematic device structure (**A**) and J–V curve (**B**) of FAMASnGeI_3_-based PSCs.

In a further stage, another absorber layer (FAMASnGeI_3_) was used for the simulation studies. The device architecture (FTO(500 nm)/ZnSe(50 nm)/FAMASnGeI_3_(500 nm)/Cu_2_O(350 nm)) of the simulated solar cells is shown in Figure 2A. The FAMASnGeI_3_-based PSCs device was simulated under the same thickness and conditions. The obtained J–V graph of the simulated device is depicted in Figure 2B. The interesting PCE of 14.25% was obtained for the simulated FAMASnGeI_3_-based PSCs device. In addition, a decent Voc of 0.83 V and an excellent Jsc of 29.05 mA/cm^2^ were obtained. The J–V graph indicated that a high Jsc value of 29.05 mA/cm^2^ could be achieved for FAMASnGeI_3_-based PSCs compared to the MAGeI_3_-based PSCs. This also suggested that FAMASnGeI_3_ has a better light absorption property, which is related to the narrow band gap of FAMASnGeI_3_.

**Figure 3:**
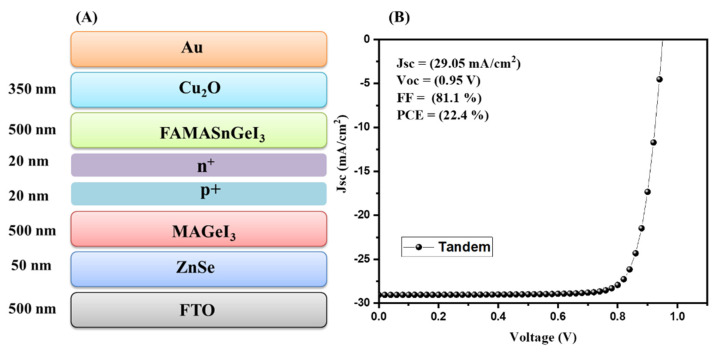
Schematic device structure (**A**) and J–V curve (**B**) of tandem with ZnSe as the ETL.

In the final step, we simulated Pb-free all-inorganic tandem solar cells using MAGeI_3_ and FAMASnGeI_3_ as the top and bottom subcells, respectively. The schematic diagram of the simulated tandem solar cell device is shown in Figure 3A. The collected J–V graph of the tandem solar cell device is shown in Figure 3B. According to Figure 3B, it can be noted that an improved PCE of 22.4% has been achieved. This revealed the potential of MAGeI_3_ and FAMASnGeI_3_ as the top and bottom subcell materials for the development of high performance Pb-free all-perovskite tandem solar cells.

**Figure 4:**
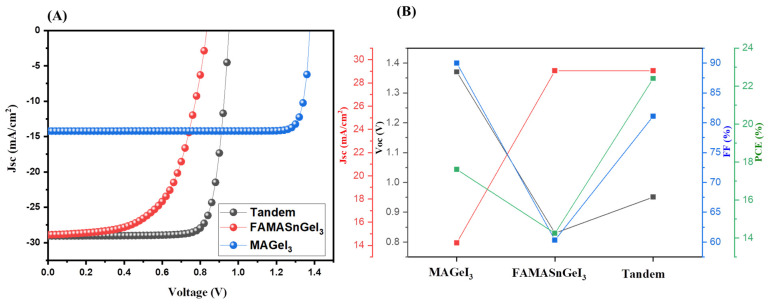
J–V curves (**A**) and photovoltaic parameters (**B**) of MAGeI_3_, FAMASnGeI_3_, and tandem PSCs using ZnSe as the ETL.

The J–V characteristic and photovoltaic parameters of the MAGeI_3_, FAMASnGeI_3_, and tandem solar cell devices are summarized in Figure 4A,B, respectively. The simulated results exhibited that the Jsc values for the FAMASnGeI_3_ and tandem devices are the same. However, different Voc values were observed for the FAMASnGeI_3_ and tandem devices.

The FAMASnGeI_3_-based PSCs device showed a lower value of Voc, whereas the highest value of Voc was observed for the MAGeI_3_-based PSCs with the lowest Jsc value. Thus, it can be clearly understood that combining the top and bottom cells improved the performance of the simulated tandem solar cells by reducing energy losses. However, MAGeI_3_ exhibited high Voc which is quite different. Further investigations and deep study are required to find out the reason behind this. In the above simulation studies, ZnSe was used as the ETL and Cu_2_O as the HTL. Thus, it is clear that the thickness of ZnSe and Cu_2_O may affect the photovoltaic performance of tandem solar cells. In this regard, we have studied the effect of the thickness of ZnSe and Cu_2_O layers. Therefore, we have investigated the influence of the thickness of ZnSe layer on the performance of tandem solar cell devices.

**Figure 5:**
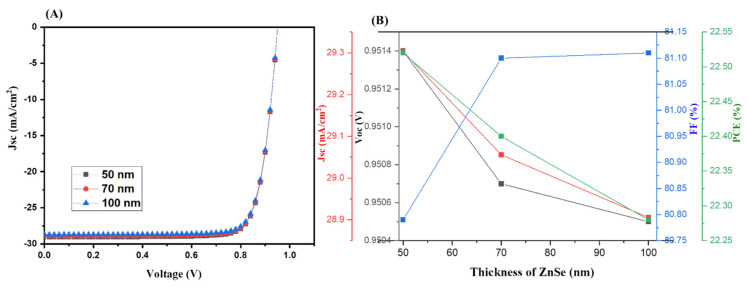
J–V curves (**A**) and photovoltaic parameters (**B**) of tandem PSCs using ZnSe (with different thicknesses) as the ETL.

We used the same thickness of 500 nm for MAGeI_3_ and FAMASnGeI_3_ layers. A thickness of 350 nm was used for the Cu_2_O layer. The thickness of ZnSe was varied in the range of 50 to 100 nm. The collected J–V graphs of the simulated tandem solar cells device at various thicknesses of ZnSe of 50–100 nm are presented in Figure 5A, whereas the extracted photovoltaic parameters are summarized in Figure 5B. The simulated results demonstrated that the PCE of the tandem solar cells decreases with increasing thickness of the ZnSe layer from 50 nm to 100 nm. It can be considered that a thin layer of ZnSe (50 nm) has better charge transport properties and enhanced photovoltaic performance compared to the 70 nm or 100 nm thick ZnSe layer. 

**Figure 6:**
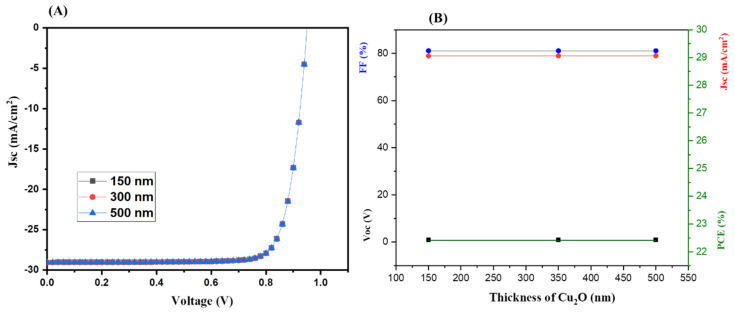
J–V curves (**A**) and photovoltaic parameters (**B**) of tandem PSCs using ZnSe as the ETL with different thicknesses of Cu_2_O.

Hence, it can be stated that the 50 nm-thick ZnSe layer is the most promising ETL, and we used this 50 nm-thick ZnSe layer for further simulation studies. Similarly, the thickness of Cu_2_O was also varied from 150 nm to 500 nm for the simulation of tandem solar cells. The tandem solar cell devices were simulated using different thicknesses of 150–500 nm of the Cu_2_O layer and the obtained results are summarized in Figure 6.

The J–V characteristics (Figure 6A) of the simulated tandem solar cells showed that the thickness of Cu_2_O HTL does not significantly alter the PCE (Figure 6B) of the tandem solar cells. Therefore, we used the optimized thickness of 350 nm for further numerical simulation studies.

**Figure 7:**
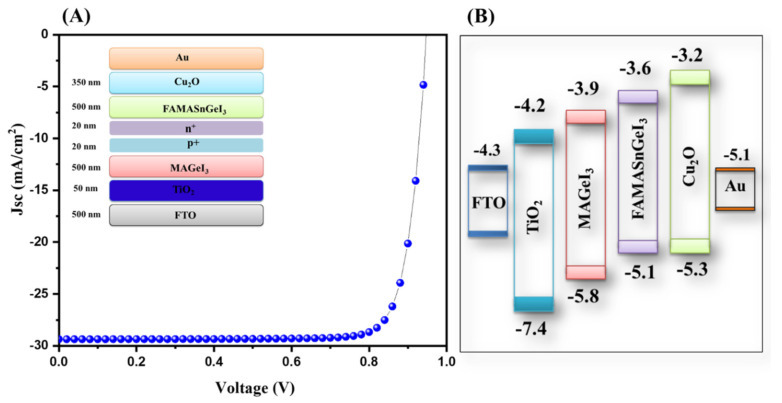
J–V curve (**A**) and energy level diagram (**B**) of tandem solar cells with TiO_2_ as the ETL.

The reported literature showed that the selection of a suitable ETL is of great significance to enhance the performance of solar cells. In this connection, we adopted different ETLs (TiO_2_, WO_3_, SnO_2_, and ZnO) for further simulation studies. The J–V characteristic curve of the TiO_2_-ETL-based tandem solar cells is displayed in Figure 7A which showed the presence of an excellent PCE of 23.18%. The photovoltaic parameters of the TiO_2_-ETL-based tandem solar cells are presented in Table S5.

**Figure 8:**
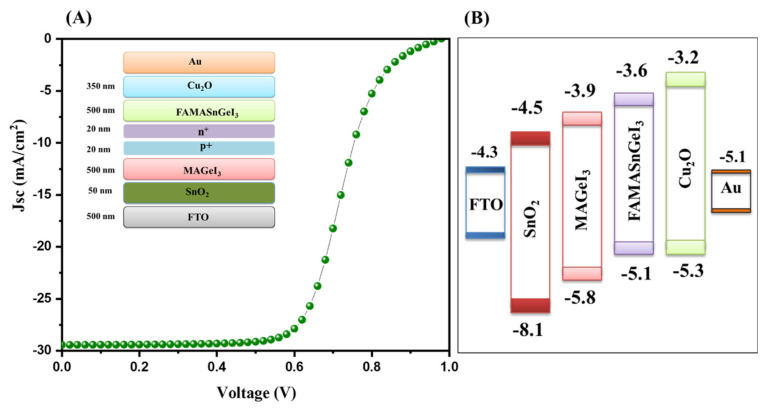
J–V curve (**A**) and energy level diagram (**B**) of tandem solar cells with SnO_2_ as the ETL.

The energy level diagram of the TiO_2_-based tandem solar cells is inserted in Figure 7B. Further, we simulated tandem solar cells using SnO_2_ as the ETL layer. The obtained results using simulation studies for SnO_2_-ETL-based tandem solar cells are presented in Figure 8A. The J–V results showed that an interesting PCE of 16.78% can be achieved using SnO_2_ as the ETL layer.

**Figure 9:**
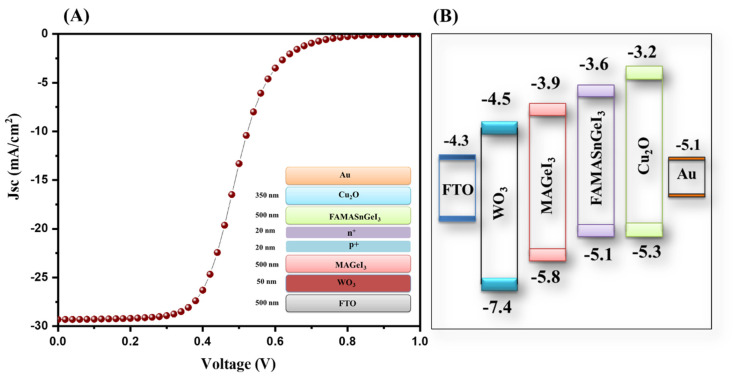
J–V curve (**A**) and energy level diagram (**B**) of tandem solar cells with WO_3_ as the ETL.

The photovoltaic parameters of the SnO_2_-ETL-based tandem solar cells are presented in Table S5. The energy level diagram of the simulated device is inserted in Figure 8B. Furthermore, a WO_3_-ETL-based tandem solar cell device was also simulated, and the J–V characteristic curve of the simulated device is presented in Figure 9A. A poor PCE of 10.52% was observed for the WO_3_-ETL-based tandem solar cells. The photovoltaic parameters of the WO_3_-ETL-based tandem solar cells are presented in Table S5. The energy level diagram of the simulated device is inserted in Figure 9B.

**Figure 10:**
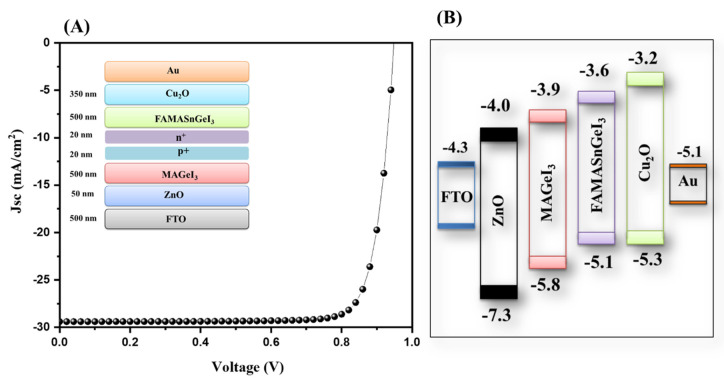
J–V curve (**A**) and energy level diagram (**B**) of tandem solar cells with ZnO as the ETL.

Furthermore, we also simulated ZnO-ETL-based tandem solar cells, and the obtained J–V characteristic data of the simulated device is presented in Figure 10A. An improved PCE of 23.11% was obtained for the ZnO-ETL-based tandem solar cells. The photovoltaic parameters of the ZnO-ETL-based tandem solar cells are presented in Table S5. The energy level diagram of the simulated device is inserted in Figure 10B.

**Figure 11:**
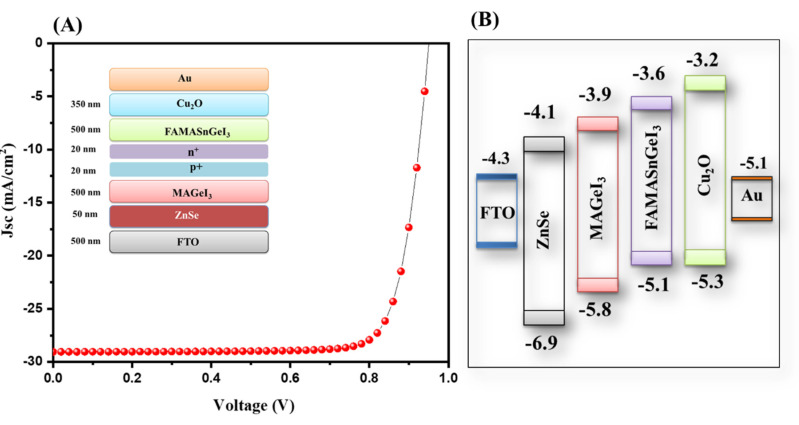
J–V curve (**A**) and energy level diagram (**B**) of tandem solar cells with ZnSe as the ETL.

The J–V characteristic of the ZnSe-ETL-based tandem solar cells is presented in Figure 11A.

The observation revealed that the highest PCE of 22.40% was obtained for ZnSe as the ETL. The photovoltaic parameters of the ZnSe-ETL-based tandem solar cells are presented in Figure 11B. The energy level diagram of the simulated device is inserted in Figure 11B. The overall observations showed that TiO_2_ is the most suitable ETL layer, whereas ZnO- and ZnSe-based tandem solar cells also exhibited excellent PCE compared to the SnO_2_- or WO_3_-based tandem solar cell devices. The photovoltaic performance of different simulated tandem solar cells is provided in Tables S3–S5. The performance of the ZnSe-ETL-based tandem solar cells is compared with previous studies in Table 1. Our obtained results are comparable with previous reports as listed in Table 1. We believe that improved PCE of all-inorganic Pb free tandem solar cells can be achieved using further novel strategies [41].

## 4. Conclusions

It can be concluded that all-perovskite lead-free tandem solar cells have been numerically simulated using SCAPS-1D. MAGeI_3_ has a wide band of 1.9 eV, which makes it a suitable candidate for the fabrication of top cells. On the other hand, FAMASnGeI_3_ has a relatively narrow band gap of 1.4 eV, and it has been adopted as an absorber layer for the simulation of the bottom cell. All-perovskite tandem solar cells were simulated using MAGeI_3_ as the top cell and FAMASnGeI_3_ as the bottom cell materials. The thickness of the electron-transport layer (ZnSe) and hole-transport layer (Cu_2_O) was optimized, and an excellent efficiency of 22.4% was obtained using SCAPS-1D. Other electron transport layers such as ZnO, WO_3_, SnO_2_, and TiO_2_ were also used, and it was observed that an improved PCE of 23.18% can be achieved using TiO_2_ as the electron-transport layer.

## Figures and Tables

**Table 1:** Comparison of tandem solar cells with previous experimental and simulated reports [15,16,17,18,19,20,21,22,37,38].

Bottom Cell	Top Cell	Jsc (mAcm^−2^)	Voc (mV)	F.F. (%)	PCE (%)	Method	References
MAPbI_3_	MAPbI_3_	6.61	1.89	56	7	Exp.	[17]
FA_0.8_Cs_0.2_Pb(I_0.7_Br_0.3_)_3_	(FASnI_3_)_0.6_(MAPbI_3_)_0.4_:Cl	14	1.92	78.1	21	Exp.	[18]
FA_0.83_Cs_0.17_Pb(I_0.5_Br_0.5_)_3_	FA_0.75_Cs_0.25_Sn_0.5_Pb_0.5_I_3_	14.5	1.66	70	17	Exp.	[19]
Cs_0.15_FA_0.85_Pb(I_0.3_Br_0.7_)_3_	MAPbI_3_	9.48	2.2	70.7	14.8	Exp.	[20]
MAPbBr_3_	MAPbI_3_	8.40	1.95	66	10.8	Exp.	[21]
CH_3_NH_3_Pb(I_0.6_Br_0.4_)_3_	CH_3_NH_3_Pb_0.5_Sn_0.5_I_3_	12.7	1.98	73	18.4	Exp.	[22]
MASnI_3_	MASnIBr_2_	13.94	1.89	60.5	15.6	Sim.	[15]
CH_3_NH_3_Pb_0.5_Sn_0.5_I_3_	Cs_2_AgBi_0.75_Sb_0.25_Br_6_	15.21	1.95	74	21.9	Sim.	[37]
FACsPb_0.5_Sn_0.5_I_3_	Cs_2_AgBi_0.75_Sb_0.25_Br_6_	14.90	1.83	63.5	17.3	Sim.	[38]
**FAMASnGeI_3_**	**MAGeI_3_**	**29.36**	**0.94**	**83.2**	**23.1**	**Sim.**	**Thiswork**

Exp. = experimental; Sim. = simulation.

## Data Availability

Not applicable.

## Supplementary Materials

The following supporting information can be downloaded at: https://www.mdpi.com/article/10.3390/nano13010096/s1, Figure S1: Flow chart for the simulation of tandem solar cells; Table S1: Numerical parameters of different materials for device simulation; Table S2: Numerical parameters of different ETLs for device simulation; Table S3: Effect of thickness of ZnSe on photovoltaic parameters; Table S4: Effect of thickness of Cu_2_O on photovoltaic parameters; Table S5: Effect of different ETLs on photovoltaic parameters. References [32,42,43] are cited in the supplementary materials.
