# Supplementary material for: Twenty-Two Percent Efficient Pb-Free All-Perovskite Tandem Solar Cells Using SCAPS-1D
Source: Nanomaterials (Basel). 2022 Dec 25;13(1):96. doi: 10.3390/nano13010096 (PMC9823386; doi:10.3390/nano13010096)
Supplement: Supplementary file 1 [file nanomaterials-13-00096-s001.zip › nanomaterials-2087368-supplementary.pdf]

Supporting Information

Twenty-Two Percent Efficient Pb-Free All-Perovskite Tandem Solar Cells Using SCAPS-1D

Ali Alsalme * and Huda Alsaeedi

Department of Chemistry, College of Science, King Saud University, Riyadh 11451, Saudi Arabia
* Correspondence: aalsalme@ksu.edu.sa

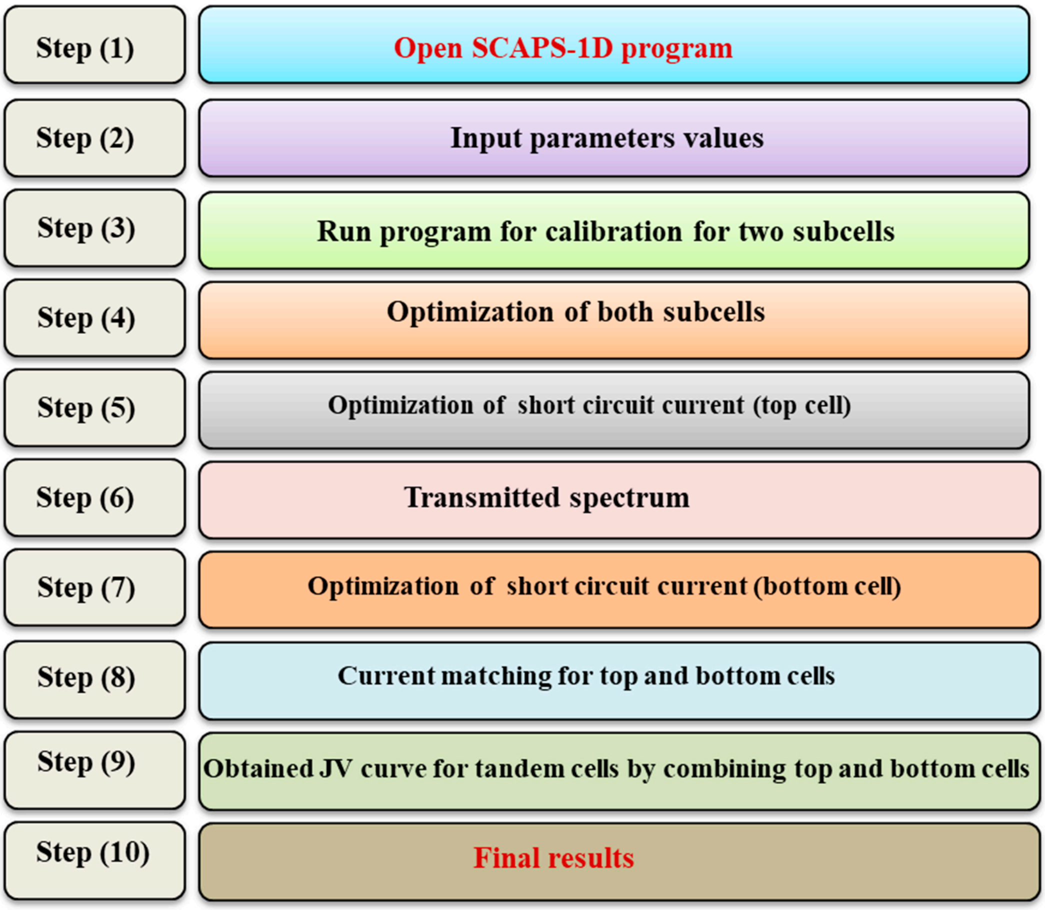

Figure S1. Flow chart for the simulation of tandem solar cells.

Table S1. Numerical parameters of different materials for device simulation.

Parameters	FTO	ZnSe	$MAGeI_3$	$FAMASnGeI_3$	Cu_2O
Thickness (nm)	500	Varying	500	500	varying
Band Gap (eV)	3.5	2.81	1.9	1.4	2.17
Electron affinity (eV)	4	4.09	3.9	3.67	3.2
Dielectric permittivity	9	8.6	10	8.2	7.11
CB effective density of states (1 cm^{-3})	2.2×10^{18}	2.2×10^{18}	1×10^{16}	2.2×10^{18}	2.020×10^{17}
VB effective density of states (1 cm^{-3})	1.8×10^{19}	1.8×10^{19}	1×10^{15}	1.8×10^{19}	1.10×10^{19}
Electron thermal velocity (cm S^{-1})	1×10^{7}	1×10^{7}	1×10^{7}	1×10^{7}	1×10^{7}
Hole thermal velocity (cm S^{-1})	1×10^{7}	1×10^{7}	1×10^{7}	1×10^{7}	1×10^{7}
Electron mobility (cm^2 VS^{-1})	20	110	16.2	20	200
Hole mobility (cm^2 VS^{-1})	10	400	10.1	20	80
Shallow uniform donor density ND (1 cm^{-3})	2×10^{19}	1×10^{18}	1×10^{9}	1×10^{13}	0
Shallow uniform acceptor density NA (1 cm^{-3})	-	0	$1 x 10^{9}$	0	1×10^{18}
Defect density (Nt)	1×10^{15}	1×10^{15}	1×10^{14}	1×10^{15}	1×10^{15}
**References**	[1]	[2]	[1]	[1]	[1]

Table S2. Numerical parameters of different ETLs for device simulation.

Parameters	TiO_2	ZnO	SnO_2	ZnSe	WO_3
Thickness (nm)	50	50	50	varying	50
Band Gap (eV)	3.2	3.3	3.5	2.81	2.92
Electron affinity (eV)	4.2	4	4.4	4.09	4.59
Dielectric permittivity	10	9	9	8.6	5.76
CB effective density of states (1 cm^{-3})	2.2×10^{18}	3.7×10^{18}	2.2×10^{18}	2.2×10^{18}	1.96×10^{19}
VB effective density of states (1 cm^{-3})	1.8×10^{19}	1.8×10^{19}	1.8×10^{19}	1.8×10^{19}	1.96×10^{19}
Electron thermal velocity (cm S^{-1})	1×10^{7}	1×10^{7}	1×10^{7}	1×10^{7}	1×10^{7}
Hole thermal velocity (cm S^{-1})	1×10^{7}	1×10^{7}	1×10^{7}	1×10^{7}	1×10^{7}
Electron mobility (cm^2 VS^{-1})	100	100	20	110	10
Hole mobility (cm^2 VS^{-1})	25	25	10	400	10
Shallow uniform donor density ND (1 cm^{-3})	1×10^{19}	5×10^{17}	2×10^{19}	1×10^{18}	3.68×10^{19}
Shallow uniform acceptor density NA (1 cm^{-3})	0	0	0	0	0
Defect density (Nt)	1×10^{15}	1×10^{15}	1×10^{15}	1×10^{15}	1×10^{15}
**References**	[2]	[1]	[1]	[2]	[3]

Table S3. Effect of thickness of ZnSe on photovoltaic parameters.

Cu_2O (nm)	$FAMASnGeI_3$ (nm)	Thickness of $MAGeI_3$ (nm)	Thickness of ZnSe (nm)	Thickness of FTO (nm)	Voc (V)	Jsc (mA/cm^2)	FF (%)	PCE (%)
350	500	500	50	500	0.9507	29.055972	81.10	22.40
350	500	500	70	500	0.9505	28.905398	81.11	22.28
350	500	500	100	500	0.9501	28.699250	81.12	22.12

Table S4. Effect of thickness of Cu_2O on photovoltaic parameters.

Cu_2O (nm)	$FAMASnGeI_3$ (nm)	Thickness of $MAGeI_3$ (nm)	Thickness of ZnSe (nm)	Thickness of FTO (nm)	Voc (V)	Jsc (mA/cm^2)	FF (%)	PCE (%)
150	500	500	50	500	0.9507	29.054281	81.10	22.40
350	500	500	50	500	0.9507	29.055972	81.10	22.40
500	500	500	50	500	0.9507	29.056919	81.10	22.40

Table S5. Effect of different ETLs on photovoltaic parameters.

Cu_2O (nm)	$FAMASnGeI_3$ (nm)	Thickness of $MAGeI_3$ (nm)	ETLs (50 nm)	Thickness of FTO (nm)	Voc (V)	Jsc (mA/cm^2)	FF (%)	PCE (%)
350	500	500	ZnSe	500	0.9507	29.055972	81.10	22.40
350	500	500	TiO_2	500	0.9478	29.362413	83.20	23.18
350	500	500	ZnO	500	0.9487	29.397456	82.85	23.11
350	500	500	SnO_2	500	0.9772	29.438141	58.34	16.78
350	500	500	WO_3	500	0.9943	29.322015	36.10	10.52

References

1. Singh, N.; Agarwal, A.; Agarwal, M. Numerical simulation of highly efficient lead-free all-perovskite tandem solar cell. *Sol. Energy* **2020**, *208*, 399–410.
2. Singh, N.; Agarwal, A.; Agarwal, M. Performance evaluation of lead–free double-perovskite solar cell. *Opt. Mater.* **2021**, *114*, 110964.
3. Otoufi, M.K.; Ranjbar, M.; Kermanpur, A.; Taghavinia, N.; Minbashi, M.; Forouzandeh, M.; Ebadi, F. Enhanced performance of planar perovskite solar cells using TiO_2/SnO_2 and TiO_2/WO_3 bilayer structures: Roles of the interfacial layers. *Sol. Energ.* **2020**, *208*, 697–707.
